# Simulation of sorghum introduction and its impacts on land use change—A case study on Lubelski region of Eastern Poland

**DOI:** 10.1111/gcbb.12669

**Published:** 2020-02-27

**Authors:** Kesheng Shu, Małgorzata Kozak, Nosra Ben Fradj, Tomasz Zylowski, Stelios Rozakis

**Affiliations:** ^1^ Department of Bioeconomy and Systems Analysis Institute of Soil Science and Plant Cultivation (IUNG-PIB) Pulawy Poland; ^2^ Centre for Energy and Environmental Management and Decision‐making (CE2MD) China University of Geosciences Wuhan China; ^3^ School of Environmental Engineering Technical University of Crete Chania Greece

**Keywords:** bioenergy, crop residues, land use, optimization, Poland, spatial agent based, sweet sorghum

## Abstract

Echoing the bioenergy development initiative in Poland, high expectations are pinned on sweet sorghum usage for biogas plants. In contrast to its high profile in the industry, the research on the introduction and production of sorghum in Poland is lagging behind. To solve this issue, in this paper we have developed a spatial‐agent dynamic model of the agricultural land use and applied the model to eastern Poland. The model suggests that the economic and technical potential of sweet sorghum in this region is 6 and 7.5 million tonnes, respectively. Its introduction process largely follows the pattern of a typical industry life cycle, with the startup at the price of 8.20 €/tonne. Along with the market penetration of sorghum, a dramatic land use change of conventional crops can be foreseen, even with a land use competition among those crops. We believe that the exploitation of unutilized agricultural land resources and improving the yield of sorghum are helpful to alleviate this land use conflict. However, a higher food demand in the future and climate change may constrain the role of sorghum. This first comprehensive and high‐resolution study to its kind in Poland can help assess the country's bioenergy policies and contribute to the development of the biogas industry.

## Graphical abstract

In recent years, biogas plants have emerged on much soil of Poland. In contrast to its high profile in the industry, our knowledge on the biomass feedstock supply for biogas production is very limited. Inspired by the successful story of Italy, we explore the feasibility of introducing sweet sorghum to this region. To accurately assess its potential and implications, we have developed a spatially agent‐based dynamic model. By providing the biomass supply curve under different scenarios, the composition of biomass feedstock as well as the corresponding land use change, we can help policy makers make informed decisions.

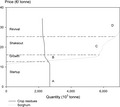

## 1 INTRODUCTION

Enhancing the share of renewable energy supply in its energy mix is one promise when Poland accessed to the EU in 2004. During 2011–2015, this share has jumped from 10.9% to 13.1%, approaching to the 2020 target of 15% set in the national “Strategy for Development of Renewable Energy” launched by the Ministry of Environment of Poland. Although much progress has been achieved so far, in the long term, there is still a long way to go, especially when this figure is compared to the EU average level of between 20.6% and 26.7% in the same period (Central Statistical Office, 2017). In view of its abundant agricultural resources, bioenergy can be a realistic and reliable source in the energy supply for Poland. However, the utilization of sugar‐based or starch‐based crops for bioenergy production, so called the first generation of biofuels, has caused serious concern about food security worldwide (Shu, Scheffran, Schneider, Yang, & Elflein, 2017).

To avert this dilemma, agricultural by‐products can be one solution. Igliński, Iglińska, Kujawski, Buczkowski, and Cichosz (2011) pinned their annual production at 25 million tonnes, with prevailing conventional crops, such as wheat, rye, and barley, contributing the majority. Rozakis, Kremmydas, Pudełko, Borzęcka‐Walker, and Faber (2013) and Scarlat, Martinov, and Dallemand (2010) conducted a similar calculation, but their special focus was placed on environmental constraints for soil conservation. Besides crop residues, energy crops are another source. In 2006, 1,000–1,500 ha of willow (Salix Viminalis) were planted in Poland, mainly located in its east and north (Ericsson, Rosenqvist, Ganko, Pisarek, & Nilsson, 2006; Nilsson et al., 2006). In 2010, 154,100 ha of land was taken by energy crops (mainly composed of energy trees and shrubs), accounting for 0.9% of the total agricultural land (i.e., 11.1% of the total arable land in Poland). Among those crops, willow was more profitable than miscanthus and triticale (Krasuska & Rosenqvist, 2012; Pudełko et al., 2012). To save arable land resources, Jezierska‐Thöle, Rudnicki, and Kluba (2016) suggested using poor soils intensively for the cultivation of energy crops.

Alternatively, sweet sorghum is another promising plant. It is originated from Africa with low soil, nutrient, and water requirements. This crop can produce grain for various purposes, whereas its parts or entire plant can be used for animal fodder, fiber, paper, building materials, and energy. Although under Polish climate conditions, it cannot produce sufficient grains, the feature of resistance to water shortages and drought, high yield of green matter makes sorghum a feasible substitution to maize, which is extraordinarily attractive in the face of climate change (Prazak, 2016). In recent years, its application for ethanol and biogas production has been widely evaluated (Agostini et al., 2016; Barcelos, Maeda, Anna, Lídia, & Pereira, 2016; Liu, Ren, Spiertz, Zhu, & Xie, 2015; Olukoya, Bellmer, Whiteley, & Aichele, 2015; Schievano et al., 2015). Its maiden application in biogas plants to replace maize silage is already under way in Poland (Igliński et al., 2012).

In contrast to its high profile in the industry, the research on the introduction and production of sorghum in Poland is much lagging behind. Although a variety of energy economy models with/without the integration of land use module, such as WEC, IIASA‐WEC, FFES, EDMONDS, and LESS/IMAGE, have been developed to simulate the bioenergy potential under different climate change scenarios (Berndes, Hoogwijk, & Broek, 2003), they aimed at weaving a general global picture rather than offering details to guide local practice in particular countries. Among rare national cases, Simon and Wiegmann (2009) developed an agricultural land use model to examine the biomass production in Germany and Eastern European Countries, where Poland is included. It estimated the country's energy potential attained from crop residues and energy crops at 590 PJ/a under the “business as usual” scenario and 540 PJ/a in the sustainable scenario. However, to the best of our knowledge, there is so far no dedicated research discussing the introduction and supply of sweet sorghum in the case of Poland. Therefore, we still do not know how the farmers will react to the market demand of sorghum and how the land use will change in a country where the agricultural sector is under reforming and restructuring. What is more important is the study can also serve as an opportunity of redesigning the supply chain of biomass feedstock in Poland as pointed out by Nilsson et al. (2006).

To fill in these gaps, we develop a spatial agent dynamic model of the agricultural land use in this paper. Different from previous empirical studies either at the state or provincial level (NUTS‐1 and NUTS‐2 level accordingly), the model is designed to delineate the introduction process of sorghum at the Local Administrative Units (LAU) level, detailing the farmers’ maneuvers and land use change as much as possible. NUTS, the abbreviation of Nomenclature of Territorial Units for Statistics, is a concept of five‐scale administrative regions defined by Eurostat. From scale 1 to 5, it represents national, voivodeship, sub‐provincial, *powiat*, and *gmina* level in Poland. Since 2017, NUTS‐4 and NUTS‐5 have been replaced by LAU. While facing the constraints of limited land resources, local climate conditions as well as the peculiar physical features of soils, farmers make decisions annually by adjusting their cultivation activities to simultaneously produce sufficient food and biomass sourced from conventional crop residues and sorghum. We opt for mimicking farmers’ decisions by means of mathematical programming, since this approach enables us to generate supply response curves using parametric optimization. The same method has been implemented in Italy for biogas chain and in Illinois, United States, for cellulosic biomass simulation (Bartoli, Cavicchioli, Kremmydas, Rozakis, & Olper, 2016; Chen & Li, 2016).

The remaining paper is structured as follows: in the next section, we build up the model and apply it to the study area, introducing the model specification and validation. Section 3 presents the simulation results of crop patterns in 2050, the supply curve of biomass feedstock, the land use competition between conventional crops and sweet sorghum, and the composition of biomass supply. Then, we discuss the features of the introduction process of sweet sorghum using the concept of industry life cycle and issues relevant to the model extension, followed by conclusions and the prospect for further research in the last section.

## 2 MATERIALS AND METHODS

### 2.1 Model construction

**Figure 1:**
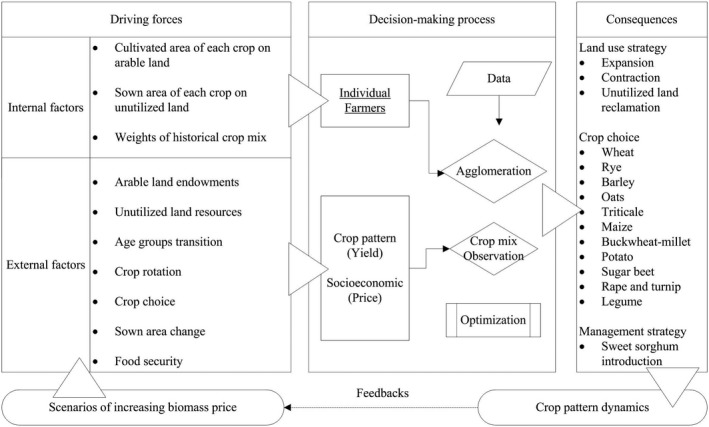
Model framework of the spatial‐agent dynamic model of agricultural land use

In this paper, we develop a mathematical optimization model for the agricultural sector to take into account the production of both food and biomass. The model framework combines the optimization approach from partial equilibrium models of the agricultural sector with historical crop‐mix approach (Figure 1). The basic simulation units are the agglomeration of individual farmers at the LAU level. In this way, the position of those farmers in the landscape and the particular soil and physiographic characteristics they own can be connected and transferred to the model. The model can, therefore, portray the heterogeneous geographical features of each unit, for example, slope, soil texture, soil depth, stoniness, watersheds, land cover, climate, and so on. Those features lead to the differentiated opportunity costs for crop cultivation, which further leaves the room for the Decision‐Making Units (DMUs) to optimizing their crop patterns.

#### 2.1.1 Optimization approach

Similar to other bottom‐up, partial equilibrium models of the agricultural sector (McCarl & Schneider, 2001; Shu, Schneider, & Scheffran, 2015), the employed optimization approach is composed of an objective function, that is, the total welfare of the agricultural sector, a set of decision variables, namely the farmers’ decisions on crop patterns, and a group of constraining equations to reflect agricultural resource endowments, technical progress, policies, and targets. Mathematically, these equations define the convex feasible region. Solving the model requires to find an optimal level for all decision variables so as to maximize the objective function and, meanwhile, subject to all constraining equations. McCarl and Spreen (1980) stated that the maximization of consumer and producer surplus, that is, the social welfare, generated the competitive market equilibrium. Therefore, the optimal levels of variables can be accounted as equilibrium levels of agricultural activities under given economic, environmental, and technological conditions. The shadow prices, derived from the marginal values of land endowments constraining equations (Equations A.2 and A.3 in Appendix A), shed lights on the opportunity cost of land resources of different soil types.

#### 2.1.2 Historical crop‐mix approach

Historical crop‐mix approach is a methodological alternative in programming models of supply response introduced by McCarl (1982). Assuming that the feasible solutions obtained from the simulation model must lie within the convex envelope of historical plantation decisions, the approach finds the best combination (i.e., weighted average) of those solutions that optimize the objective function under the prevailing market conditions. In such a way, modelers do not need document full information about microlevel input and output data and extreme points of the individual firm problems, which are nearly infeasible to collect from the field, to exhaustively depict the farmer’ decision‐making process. Instead, modelers usually have the access to the observed historical crop patterns (or crop mix) from publicly available statistics and other data sources, which already reflect the aggregation of the optimum responses of individual firms, taking into consideration agronomic crop sequence restrictions, risk diversification, avoidance of high fluctuations in labor and machinery demand, and so on. This approach is justified by Önal and McCarl (1989, 1991). They explained that the optimum solutions of a staircase linear program, including all firms as independent decision makers, are in a one‐to‐one correspondence with the optimum solutions of the individual firm models. Therefore, the aggregate solution (i.e., mathematically, an extreme point of the aggregate model‐assuming linear constraints) is formed by stacking the optimum solutions (i.e., extreme points) of the firm level models (Chen & Önal, 2012). Although having advantages of computing conveniently, unlimited access to aggregate data, and replacing subjective decision‐making constraints with objective ones, this approach has difficulty on predicting the plantation of new crops, as the crop pattern of these crops is excluded from historical crop mix. To fix this issue, the crop‐mix approach should be combined with other modeling approaches to limit the flexibility, such as sown area change constraints and crop rotation activities illustrated in Equations (A.6) and (A7–A12) in Appendix A, respectively.

### 2.2 Model structure

The optimized agricultural sector model is a regional recursive dynamic partial equilibrium model illustrating the land use change in the face of food security and biomass demand. The linear programming model is coded in the commercial software GAMS, using CPLEX as a solver.

We develop a modeling framework to mimic the annually recurring decision‐making process of farmers, the endogenous agents of the model (Figure 1). Aiming at limiting the number of farmers to the computable level, we aggregate all individual farmers in one *gmina* and treat it as one agent in our regional agricultural sector model. In this way, 213 agents are set in our model. They decide on the type and sown area of crops as well as the levels of relevant cropping activities. Throughout the model time span of 2018–2050, those decisions made in 1 year are consistently transferred into the next year, introducing recursive dynamics into the model. The farmers are expected to react iteratively to the changing market price of biomass by adjusting their land use, that is, crop patterns and the plantation of sweet sorghum up to the point at which the total welfare is maximized. The exogenously represented actors in the model are biomass consumers and the government via the setting of external factors, for example, the biomass price and food demand, the subsidy level toward food production and crop plantation.

Our model determines the optimal allocation of arable and unutilized land resources with different soil types among conventional crops and sweet sorghum to simultaneously meet the demand for food and react to the signal of biomass price (Table 1). In this empirical research, 15 locally prevailing conventional crops are covered. They are winter wheat, spring wheat, rye, winter barley, spring barley, oats, winter triticale, spring triticale, maize for grain, maize for forage, buckwheat millet and other, potatoes, sugar beet, rape and turnip rape, leguminous edible, whose cultivation area accounts for above 80% of the total available arable land. Particularly, sweet sorghum, representing for dedicated energy crop (DEC), is examined. In mathematical terms, the model comprises an objective function, 1,015,594 single decision variables, and 678,169 single constraining equations (for the elements of specific indices, please refer to Appendix A).

**Table 1:** The description of model equations and variables

Model equation	Mathematical structure	Number	Description
Objective function	WELFARE=REVENUE-COST	Equation (1)	The sum of producer revenue in all commodity markets, minus specific and unspecific cultivation cost.
Physical constraints	$LAND^{\mathrm{concrop}}+LAND^{\mathrm{enecrop}}\leq endowments^{\mathrm{arableland}}$ $UNULAND^{\mathrm{concrop}}+UNULAND^{\mathrm{enecrop}}\leq endowments^{\mathrm{unutilizedland}}$ $LAND^{\mathrm{enecrop}}\leq \alpha \cdot endowments^{\mathrm{arableland}}$	Equation (2)	The cultivated land in each region and time period cannot exceed given endowments.
	$LAND^{\mathrm{concrop}}+UNULAND^{\mathrm{concrop}}\leq \gamma \cdot croppattern_{\mathrm{his}}$	Equation (3)	Linking projected sown area of each crop in the future to its historical crop pattern.
Technical constraints	$LAND_{a}^{\mathrm{concrop}}\leq \beta \cdot LAND_{b}^{\mathrm{concrop}}$	Equation (4)	Obeying the practice of crop rotations, the sown area of main crop is fixed to the area of its pre‐crop and post‐crop.
Policy constraints	$demand^{\mathrm{food}}\leq yield^{\mathrm{food}}\times LAND^{\mathrm{concrop}}$	Equation (5)	Food production needs to satisfy minimum food demand.
	$\sum_{\mathrm{his}}\left(landuse_{\mathrm{his}}^{\mathrm{concrop}}\times CMIXP_{\mathrm{his}}\right)=LAND^{\mathrm{concrop}}$	Equation (6)	Cropping activities are shaped by historically observed choices to ensure CAP diversification rules.
	$\lambda \cdot \sum_{early\;his}CMIXP_{\mathrm{his}}-\sum_{recent\;his}CMIXP_{\mathrm{his}}\leq 0$	Equation (7)	The observations in recent years play a more important role than early years in predicting the cropping activities in the future.
Decision variables	$\begin{array}{c} LAND^{\mathrm{concrop}},\;LAND^{\mathrm{enecrop}},\\ UNULAND^{\mathrm{concrop}},\;UNULAND^{\mathrm{enecrop}} \end{array}$		Cultivated area includes arable land and unutilized land. Crops in the model are divided into conventional crops and energy crops.
	$CMIXP_{\mathrm{his}}$		Weights of historical crop patterns for projections.

The objective function Equation (1) (Equation (A.1) in Appendix A) maximizes the present value of the total welfare of agricultural sector over a 33 year horizon with an annual step. It is calculated by subtracting the total costs from the total revenues. Total revenues come from the sales and governmental subsidies of two types of commercial products under current and envisioned framework of Common Agricultural Policy (CAP) until 2027: grains from conventional crops (Line 2 and 5 in Equation (A.1) and biomass feedstock from crop residues of conventional crops and sweet sorghum (Line 3, 4, and 6 in Equation (A.1). Since crop residues are a by‐product of grains, the revenue from their sales is excluded from our accounting. Total costs are caused by agricultural activities and input factors invested in crop cultivation.

Values of the decision variables, describing the agglomerate farmers’ cropping activities (internal factors in Figure 1), are endogenously determined by the optimization process. The constraining equations illustrate the factors influencing the agents’ decision‐making process (external factors in Figure 1). Mathematically, they define the convex feasible region for all decision variables. In this model, they can be categorized into three groups: physical, technical, and policy restrictions.

Physical restrictions are constructed to guide the allocation of land resources. Equation (2) regulates the allocated arable and unutilized land resources for food and biomass production be within the range of their endowments. Equation 3 links the projected sown area of each crop in the future to its historical crop pattern at the LAU level. Technical restrictions (Equation 4) are applied to guarantee the consistency of sown areas between pre‐crop, main crop, and post‐crop involved in the same crop rotation. Policy restrictions here refer to the policy targets of maintaining food security (Equation 5) and of carrying out CAP (Equations 6 and 7). Specifically, the crop mix in the projection should be a linear combination of historical observations in accordance with the principle of the historical crop‐mix approach. A detailed description of our model is given in Appendix A.

### 2.3 Model application

#### 2.3.1 Case study area

**Figure 2:**
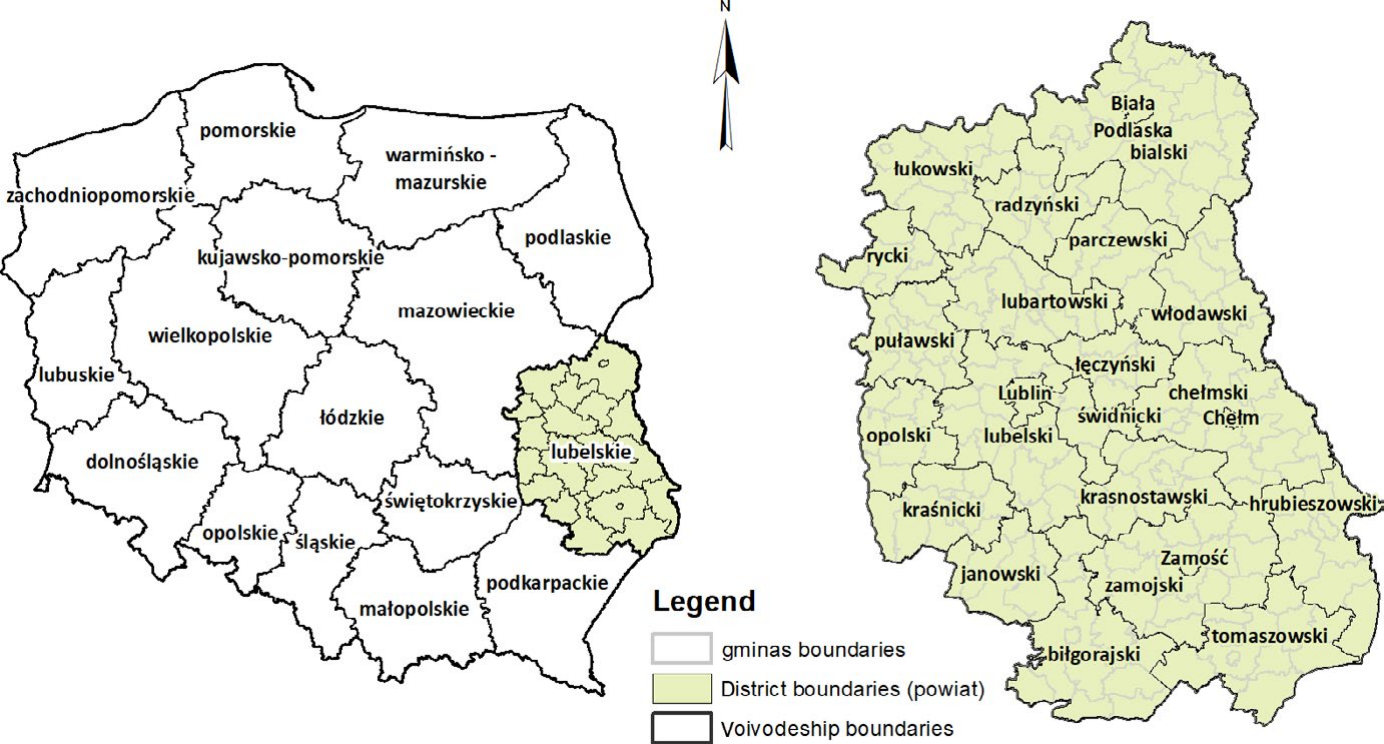
Location of Lubelski voivodeship in Poland

Lubelski voivodeship, a well‐recognized important agriculture base for Poland, locates on the southeast of the country (Figure 2). Its share of agricultural land in total land is up to 70%. Thanks to the favorable soil quality, water conditions, agro‐climate and terrain, the voivodeship, as a whole, ranks third in Poland in terms of natural resources potential, although heterogeneous soil qualities in the province can be observed. The best soils are located in Lublin *powiat* near Nałęczów and in Hrubieszowski *powiat*, while the worst are scattered in Włodawski, Bialski *powiat*, near Parczew, Lubartów, Biłgoraj, and eastern part of Łukowski and Janowski *powiat*. In 2017, the total output of cereal crops amounted to 3,641 thousand tonnes, contributing 11.4% of the total grain harvest, ranking the second in Poland. Besides cereal crops, Lubelski voivodeship is also a leader in the plantation of many cash and horticulture crops (with the share to the domestic production in 2017 listed in parenthesis, respectively), such as sugar beets (17.7%), vegetables (11%), fruits from trees (13.2%), shrubs and berries (45.6%), hops (90%), and tobacco (65%; Statistics Poland, 2019).

#### 2.3.2 Model specifications

##### Source of biomass feedstock

Along with the evidence from Italian case that the combination of DEC with crop residues provides more efficient input in the biogas chain (Schievano et al., 2015), we treat sweet sorghum and crop residues from conventional crops as two main sources of biomass feedstock in our model. After setting widely accepted dry weight straw/grain ratios to each conventional crop (Table 2), we also take into consideration the use of crop residues for improving soil organic matter (SOM; Table 3).

**Table 2:** Dry weight ratio of straw to grain for different crops

Crop	Ratio	Crop	Ratio	Crop	Ratio
Wheat	1.50	Triticale	0.65	Sugar beet	0.25
Rye	1.50	Maize for grain	1.00	Potatoes	0.25
Barley	1.50	Maize for forage	0.00	Rape and turnip	1.56
Oats	1.00	Buckwheat, millet, and other	1.50	Leguminous edible	1.00

**Table 3:** Utilization ratio of crop residues for bioenergy purpose after soil organic matter improvement

Crop	Ratio	Crop	Ratio	Crop	Ratio
Wheat	0.60	Triticale	0.50	Sugar beet	0.67
Rye	0.60	Maize for grain	0.50	Potatoes	0.50
Barley	0.60	Maize for forage	0.00	Rape and turnip	0.50
Oats	0.60	Buckwheat, millet, and other	0.50	Leguminous edible	0.50

##### Historical crop patterns (crop mix)

Generally, the historical crop patterns are collected from the official database maintained by the Central Statistical Office of Poland and its branch located in Lubelski voivodeship. In terms of the data resolution, they can be further divided into two layers.

**Figure 3:**
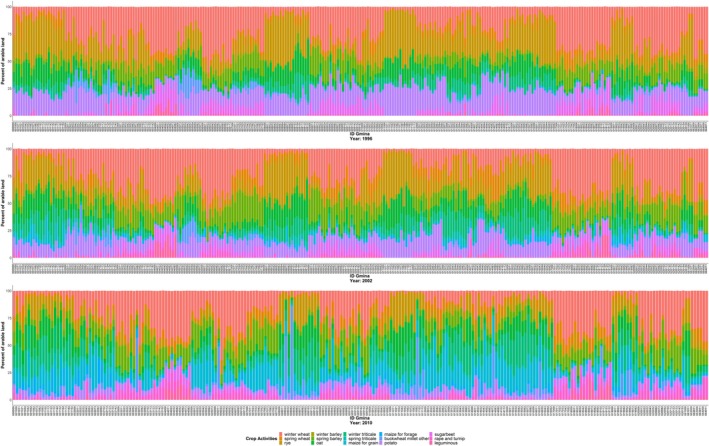
Historical crop patterns in Lubelski region at LAU level. Data source: Statistics Poland (2019)

One layer has a finer resolution and is reported at the LAU level. Those data were collected in the National Agricultural Census 1996, 2002, and 2010 (Figure 3). Against the survey data in 1996 and 2002, the data in 2010 can reflect recent trends better, due to the Poland accession to the EU in 2004. Therefore, we use the historical crop pattern in 2010 at the LAU level as a benchmark to speculate the agricultural activities of each DMU in the future years (Equation 3).

**Figure 4:**
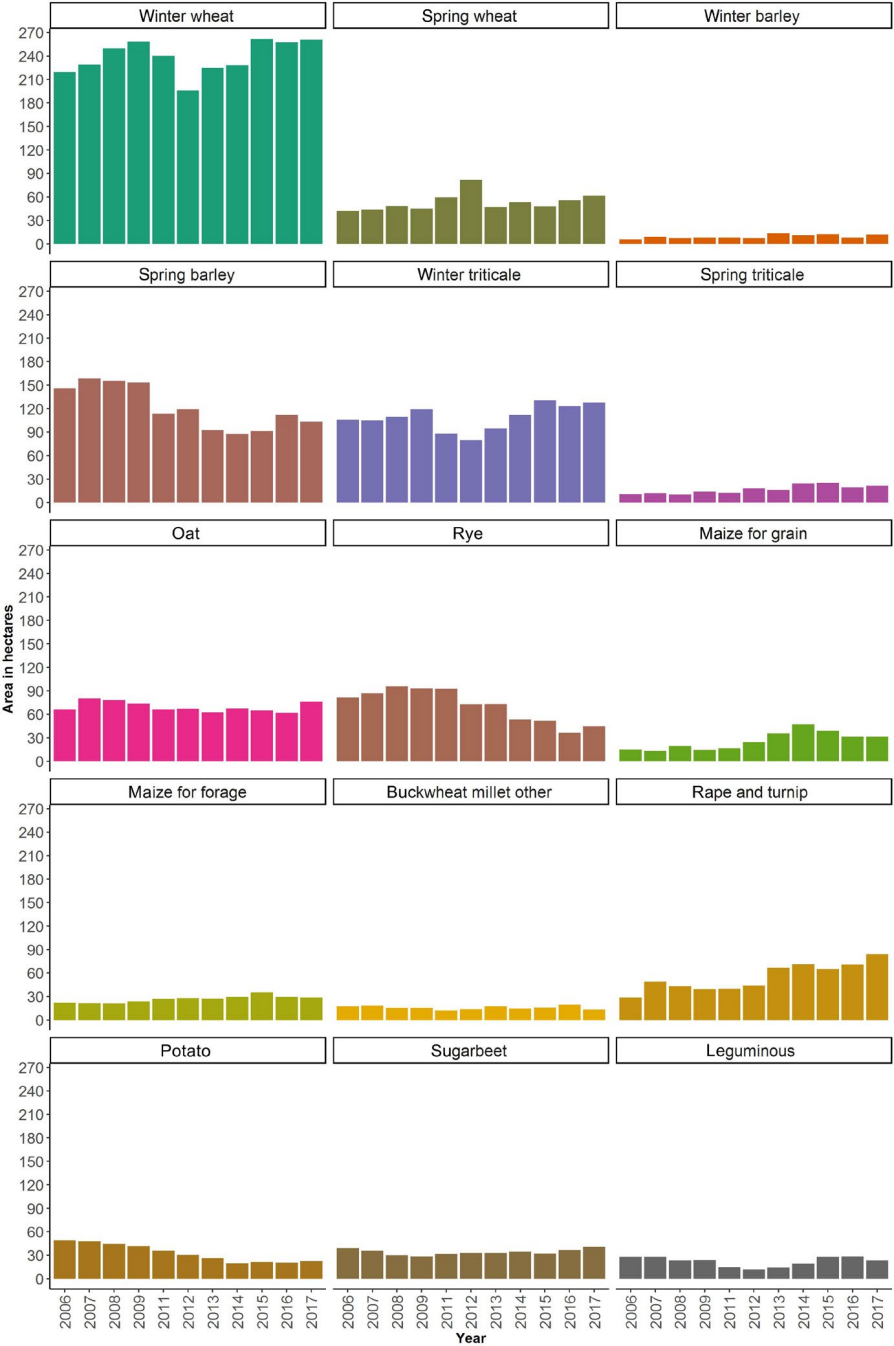
Historical crop patterns in Lubelski region at NUTS‐2 level Data source: Statistics Poland (2019)

The other layer reports crop patterns in the years of 2006–2009 and 2011–2018 at the NUTS‐2 level (Figure 4). The data of 2006–2009 and 2011–2017, which deliver the response of individual farmers to the physical, technical, and policy constraints, are picked out to define the relative share of each conventional crop in every historical crop mix (Equation 6). In order to enhance the high influence of recent years’ observations on the model projection, we assign the weights of crop patterns in 2013–2017 twice as much as in 2006–2009 and 2011–2012 (Equation 7). For the data of 2018, it is separately used to validate the model by the comparison with simulation results for the same year.

##### Agricultural land resources and soil types

As we do not include all crops currently planted in the Lubelski voivodeship in our model, using the statistical data on the arable land endowments of each *gmina* will inevitably overestimate the total sown area of the examined 15 crops. To estimate the arable land potential for those 15 crops accurately, we first calculate their aggregate sown area in 1996, 2002, and 2010 based on historical crop patterns at the LAU level. Then, we deliver the maximum value among the 3 years to the model as the upper limit of the available arable land endowments.

In terms of the unutilized agricultural land resources as well as the share of four soil types, that is, very light, light, average, and heavy, we pick data from the Agricultural Database of Institute of Soil Science and Plant Cultivation. An example is presented in Table 4. These four soil types are categorized based on the granulometric composition in the soil profile (i.e., the determination of particle size of subsoil). Due to the fact that crops on heavy and average soil type usually have similar yields, we combine the two categories in the research.

**Table 4:** An exemplary case of land endowments and soil types at Local Administrative Units level

*Gmina* ID	Arable land resources				Unutilized land resources			
	Total area (ha)	Very light (%)	Light (%)	Heavy & average (%)	Total area (ha)	Very light (%)	Light (%)	Heavy & average (%)
0601011	895.98	29.01	58.76	12.23	186.62	51.20	38.76	10.04
0601021	447.82	5.88	7.35	86.77	83.41	6.14	10.26	83.60

##### Crop rotation and crop yield

The information on crop rotations and their occurrence on relevant soil types is also from our own database (Table 5).

**Table 5:** Observed crop rotations in the Lubelski voivodeship^a^

Soil type	Crop rotations
Heavy & average	½ sugar beet, spring wheat, winter wheat, winter barley ½ maize^b^, spring wheat, winter wheat, winter barley
	½ leguminous edible, winter wheat, winter wheat, winter barley ½ rape and turnip, winter wheat, winter wheat, winter barley
Light/heavy & average	Potatoes, spring barley, winter triticale, cereal mixture, winter triticale
Very light/light	Oats, rye, cereal mixture

a Data from original report (Matyka et al., 2011) elaborated and updated by the first author M. Matyka (personal communication, December 2, 2019), an expert in systems and economics of local crop production.

b Maize includes maize for grains and maize for forage; cereal mixture includes oats, spring barley, and spring wheat.

The average crop yields of conventional crops are from the database of the Central Statistical Office of Poland. To reflect the uncertainty of climate change impacts, we suppose the variation of crop yields in the projection obeys a normal distribution with the mean value identical to their benchmark value in 2010. Furthermore, aiming at differentiating the effects of crop rotations and soil types, we introduce the following assumptions: (1) Crop rotations and monoculture are allowed to occur on less preferred soil type, which is one level upward, that is, from “very light” to “light” and from “light” to “heavy & average” soil type. (2) The average crop yields are achieved under the condition of preferred soil type and monoculture. When the crop is planted on (a) preferred soil type and crop rotation or (b) less preferred soil type and monoculture, or (c) less preferred soil type and crop rotation, the yield will increase 10%, decrease 10%, and be unchanged, respectively. (3) Sweet sorghum can only be planted monoculturally on heavy & average soil with green mass yield of 50 tonnes/ha and dry mass yield of 20 tonnes/ha (Księżak, Matyka, Bojarszczuk, & Kacprzak, 2012; Lal, 2005). In the sensitivity analysis, the only DEC is also allowed to be involved in crop rotations.

##### Source of biomass feedstock

In our model, two sources of biomass feedstock are considered. The first source is crop residues. Since they are by‐products, in the model we allocate their production cost to the main products, for example, grains. Therefore, we assign a zero market price to crop residues.

The second one is sweet sorghum. Different from conventional crops, it is a dedicated energy crop for biomass production. To compensate its production cost, we introduce reasonable market prices. In order to reveal how sensitive the farmers are to the price signals of sweet sorghum, we set up 30 scenarios to simulate the price change ranging from 0 to 34.5 €/tonne with a step of 1.17 €/tonne. Additionally, we impose a limitation of 20% on the share of projected sown area of sweet sorghum to the total arable land of high‐average soil type. This assumption is to follow farmers’ conservative attitude toward new cultivars.

##### Food demand and food trade

Demand functions for domestic consumption and for exports and imports of tradable commodities are specified for individual commodities by linking the parameter of self‐sufficient ratio to their average output in recent years. This ratio is set at 75% in the benchmark after considering the local practice. However, it is possible to shift these demand functions upward to allow for increasing demand for food over time. In the sensitivity analysis, we will increase the self‐sufficient ratio by 5% to explore such a possibility.

#### 2.3.3 Validation

**Figure 5:**
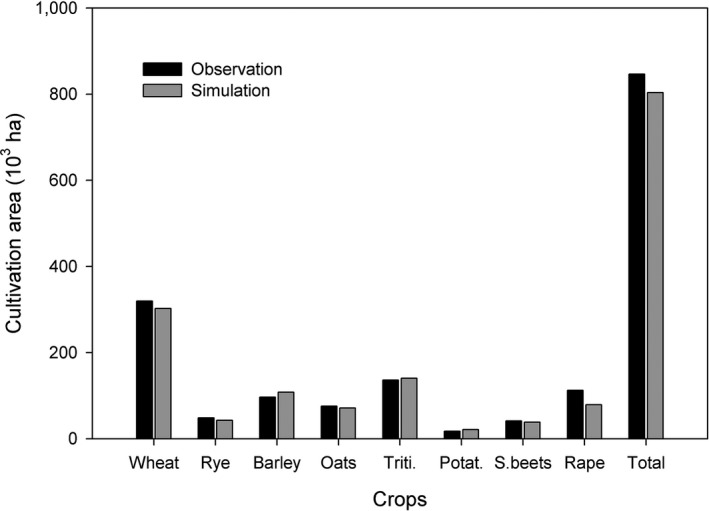
Comparison between observation and simulation of crops cultivation area in the year of 2018 (Unit: 10^3^ ha)

We run the benchmark model without the introduction of sweet sorghum to test the accuracy of the simulation for 2018 against the observation in the same year. Although the model has a finer resolution to report the cultivation area of each crop at the LAU level with the information on associated crop patterns and soil types, we have to aggregate it to the provincial level so as to match the observation. The observation and simulation results are listed in Figure 5.

Our model presents a reliable ability of replicating the patterns of prevailing cereal crops. Among wheat, rye, barley, oats, triticale, and sugar beets, the gap between observation and simulation stands well below 12%. As for potatoes and rape and turnip, it can restore about 75% of the observation. The volatile cultivation of the two crops (e.g., the sown area of potatoes and rape and turnip changes between 19.45–35.79 and 40.12–84.13 thou. ha in 2011–2017 individually, see Figure 4) has largely hampered accurate prediction. In total, the model can explain 95% of the crop pattern on the provincial level, which lays a solid foundation for our following analysis.

#### 2.3.4 Scenarios setting for sensitivity analysis

Given the relatively long projection timeframe and the wide spectrum of our simulation, high uncertainties can be widely expected in the assumptions adopted in our model. In order to test the model robustness as well as enhance our understanding of the impacts of those factors, we pick out several critical parameters and set four scenarios as follows. In the first “tight food trade scenario,” we assume a more stringent food import and export situation, where the food self‐sufficient ratio of the examined region will increase from 75% to 80%, meaning local production needs to satisfy up to 80% of the food demand. The second “optimistic climate change scenario” is created to assume an adaptation pathway to climate change employed in the agriculture sector in Poland, such as the changes of agro‐technical practices, the introduction of new cultivars, protection of soil and water resources, and so on. Thanks to those measures, the yields of conventional crops will only drop 5% and of sweet sorghum will maintain. On the contrary, poles in the “pessimistic climate change scenario” are aware of the climate change issue, but do not consider it as a priority issue. The agriculture sector hesitates to take any adaptive measures. As such, the yields of conventional crops and sweet sorghum will decrease by 15% and 5% separately. The last scenario “sorghum in crop rotation” examines the plantation of sorghum in crop rotations. Although there is no such practice so far, we assume it can replace maize and be planted in the rotation of “sorghum, spring wheat, winter wheat, winter barley” on the “heavy & average” soil. In such a case, its yield will be 5% higher than its monoculture counterpart.

## 3 RESULTS

### 3.1 Benchmark: Crop pattern in 2050

**Figure 6:**
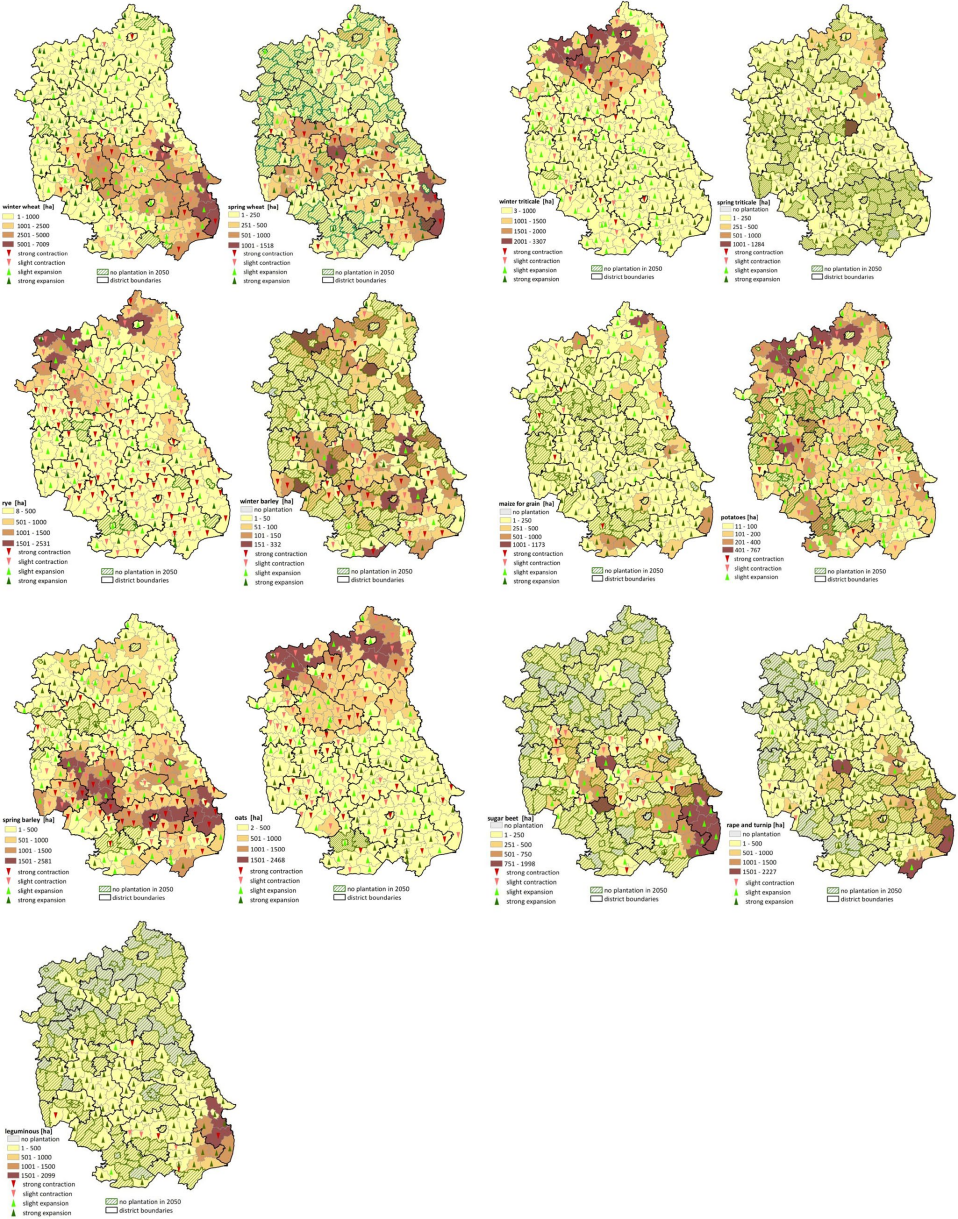
Arable land use change in Lubelski voivodeship between 2010 and 2050. Notes: Polygons with classified colours denote the cultivation area of each crop at LAU level in 2010. Arrows demonstrate the direction of land use change. When the sown area in 2050 decreases 50% and above compared to 2010, it is identified as strong contraction. When the area doubles, it is identified as strong expansion. The crops of maize for forage and buckwheat millet and other are not included due to their data vacancy in 2010

Figure 6 presents the cultivation area of each crop in 2010 and its projected change between 2010 and 2050 on arable land. As one of the main cereal crops, the cultivation of winter wheat is expected to expand in most areas. While northern *powiat*s will increase significantly, the southeastern ones, a traditional base for this crop plantation only expects a mild growth. For spring wheat, its cultivation area will contract dramatically. Although the northeastern *powiat*s will see a moderate decrease, the strong contraction will occur in the south‐eastern *powiat*s, where the crop was largely planted in 2010. The same trend can be observed in the north‐western and southern *powiat*s for winter barley, except that a strong expansion will occur in the north‐eastern and south‐eastern *powiat*s. The cultivation area of rye and oats will decrease mainly in the northern and central *powiat*s, where they were planted in a large scale in 2010. However, in comparison with rye, we can observe the slight increase of oats cultivation in the vast area of southern *powiat*s. Spring barley shows a very different trend. Its cultivation will expand slightly in northern *powiat*s, while its strongholds in 2010 will face shrinkage. Winter triticale and spring triticale share the similar pattern to some extent. Strong expansion is the mainstream tune for both crops. However, our model suggests more *powiat*s to quit from the spring triticale cultivation. This arrangement also holds true for the plantation of maize for grain.

As to non‐cereal crops, the model advices us to concentrate their sown areas. Potatoes should be planted in northern and southern part, sugar beets and leguminous edible in central and south‐eastern part, and rape and turnip in northern and south‐western part.

In terms of the agronomic practice in 2050 (Table 6), crop rotations will be popular on arable land with very light soil. Among six typical crop rotations, the “oats, rye, cereal mixture” and “potatoes, spring barley, winter triticale, cereal mixture, winter triticale” rotations will be practiced most frequently. On unutilized agricultural land, monoculture will be more welcomed, as these candidate land resources provide farmers more flexibility in crop pattern decisions.

**Table 6:** Simulated agronomical practice in 2050 (Unit: 10^3^ ha)

Soil type	Agronomical practice		Arable land	Unutilized agricultural land
Heavy & average	Crop rotation	s‐sw‐ww‐wb	0.51	0.22
		m‐sw‐ww‐wb	30.41	1.93
		l‐ww‐ww‐wb	2.32	1.14
		ra‐ww‐ww‐wb	0	0.14
		p‐sb‐wt‐c‐wt	40.08	1.71
	Monoculture		482.58	36.79
Light	Crop rotation	p‐sb‐wt‐c‐wt	91.44	2.70
		o‐ry‐c	4.38	0
	Monoculture		96.87	18.76
Very light	Crop rotation	o‐ry‐c	129.35	2.30
	Monoculture		17.90	35.92
Total cultivation area			895.85	101.63

Notes

Abbreviations of crop rotation patterns are as follows: “s‐sw‐ww‐wb” refers to “sugar beet, spring wheat, winter wheat, winter barley”; “m‐sw‐ww‐wb” refers to “maize, spring wheat, winter wheat, winter barley”; “l‐ww‐ww‐wb” refers to “leguminous edible, winter wheat, winter wheat, winter barley”; “ra‐ww‐ww‐wb” refers to “rape and turnip, winter wheat, winter wheat, winter barley”; “p‐sb‐wt‐c‐wt” refers to “potatoes, spring barley, winter triticale, cereal mixture, winter triticale”; “o‐ry‐c” refers to oats, rye, cereal mixture.

### 3.2 Introduction of sweet sorghum

#### 3.2.1 Biomass supply curve

**Figure 7:**
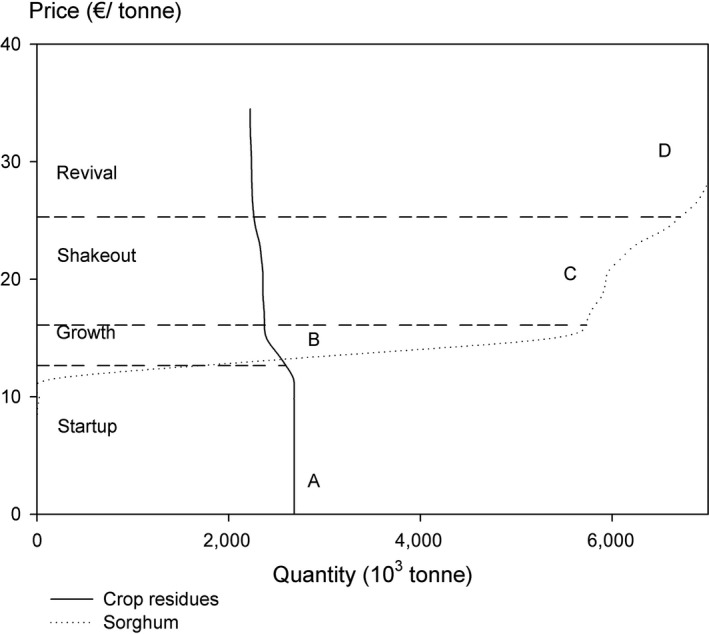
Biomass supply curve composed of crop residues and sorghum with four illustrative points. Notes: The pairs of price‐quantity at points A, B, C, D are 0 €/tonne, 2,684 thou. tonne; 12.65 €/tonne, 4,308 thou. tonne; 16.10 €/tonne, 8,106 thou. tonne; 25.30 €/tonne, 8,975 thou. tonne

Under each market price of sweet sorghum, we retrieve the corresponding output of biomass from crop residues and sorghum simultaneously from the model simulation. Figure 7 presents all observed pairs of biomass price and quantity supplied, with each pair of price–quantity illustrating the price level at which the DMUs are willing to supply corresponding quantity of biomass. At low levels of market price, specifically between 0 and 8.20 €/tonne in our case, crop residues from conventional crops provide the full supply (point A in Figure 7). As we have pointed out, the by‐product feature of crop residues enables their supply at zero price. Due to the extra investment required for the sorghum plantation, its introduction process does not take off until the market price climbs up to 8.20 €/tonne. In the range of between 8.20 and 9.40 €/tonne, the sown area of sorghum grows very slow. Once its price touches the line of 11.70 €/tonne, its cultivation gains full momentum, while the output of crop residues undergoes a relatively slight decrease (compare point B and C in Figure 7). When biomass price shifts from 15.20 to 16.40 €/tonne, the supply curve of crop residues and sorghum experiences a turning point successively. Above the turning points, both curves are insensitive to the stimulus of price, implying the exhaustion of arable land resources (point C in Figure 7). However, once the price continues to increase up to 21.10 €/tonne, which brings sufficient financial payback to unutilized agricultural land reclamation, the supply curve of sorghum restores elasticity (point D in Figure 7).

#### 3.2.2 Sensitivity analysis

**Figure 8:**
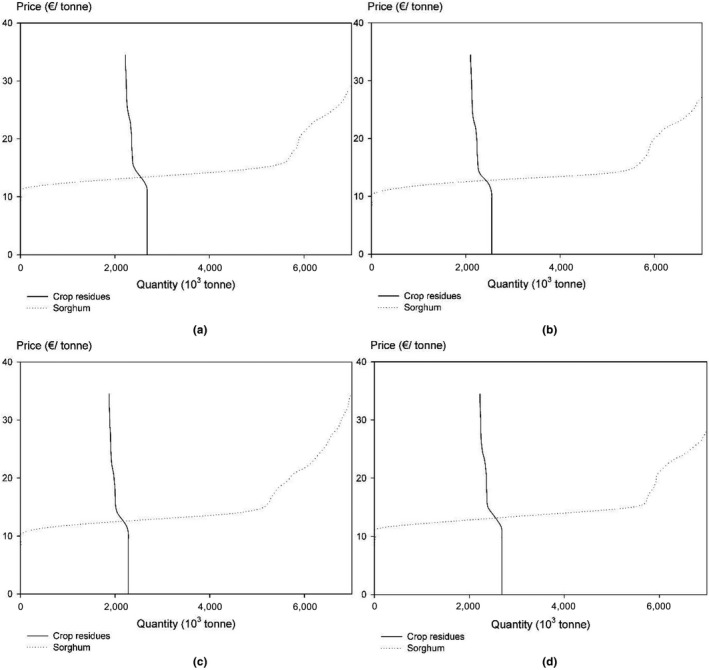
Sensitivity analysis of the supply curve under (a) tight food trade (b) optimistic climate change (c) pessimistic climate change (d) sorghum in crop rotation

The biomass supply curve under each scenario is presented in Figure 8. Meanwhile, the corresponding allocation of arable land and unutilized agricultural land resources between conventional crops and sweet sorghum is provided in Table 7. It is interesting to see that the supply curve in each scenario generally shares a similar pattern to the base case, implying the robustness of the model results in the presence of uncertainty.

**Table 7:** Usage of arable land and unutilized agricultural land resources under different scenarios (Unit: 10^3^ ha)

Scenario			Biomass price (€/tonne)	Base case	Tight food trade A	Optimistic climate change B	Pessimistic climate change C	Sorghum in crop rotation D
Arable land resources	Conventional crops	Crop rotation	12.65	293.13	293.75	290.01	293.35	292.14
			16.10	276.89	278.21	278.71	284.09	276.87
		Monoculture	12.65	569.82	574.85	561.62	554.23	569.81
			16.10	508.38	508.61	506.99	504.03	508.41
	Sorghum	Crop rotation	12.65	—	—	—	—	—
			16.10	—	—	—	—	—
		Monoculture	12.65	33.90	27.25	44.22	48.26	33.90
			16.10	110.58	109.03	110.15	107.73	110.58
Unutilized agricultural land resources	Conventional crops	Crop rotation	12.65	9.30	9.70	9.98	13.73	9.30
			16.10	6.73	9.60	8.23	14.87	6.73
		Monoculture	12.65	92.08	91.74	91.08	87.45	92.07
			16.10	90.97	88.64	89.05	83.69	90.97
	Sorghum	Crop rotation	12.65	—	—	—	—	0.00^a^
			16.10	—	—	—	—	0.00^b^
		Monoculture	12.65	0.32	0.30	0.60	0.47	0.32
			16.10	4.10	3.60	4.51	3.22	4.09

a This table shows a value with only two decimal places. The value of 0.00 here actually refers to 4.56 ha sown area of sorghum.

b The actual sown area is 2.41 ha.

In tight food trade scenario (Figure 8a), higher domestic food demand brings out the larger sown area of conventional crops and higher output of crop residues at the cost of a shrinking sorghum supply. This result performs most significantly when the biomass price climbs up from 11.50 to 16.10 €/tonne. In comparison with the base case, the supply curve of crop residues and sorghum shifts inward in two climate change scenarios (Figure 8b,c). This dwindling supply, which is heavily plagued by the crop yield loss, suggests that climate change may deteriorate the land use competition between conventional crops and sorghum. In view of the involvement of sweet sorghum in crop rotations, no significant changes in biomass supply can be observed (Figure 8d). The “sorghum, spring wheat, winter wheat, winter barley” rotation is only expected to appear on unutilized agricultural land resources to a very limited extent (Table 7).

In the following subsections, we will present the land resources allocation and the composition of biomass supply at those four characteristic points so as to dynamically describe the introduction process of sweet sorghum.

#### 3.2.3 Land resources allocation

The introduction of sweet sorghum strongly influences the land use pattern of arable land and unutilized agricultural land (Table 8). In terms of the heavy & average soil on which sweet sorghum is cultivated, a shift in the agronomic practice from crop rotation to monoculture occurs, paving the way for sorghum plantation. Among monoculture crops, the sown area of sorghum expands rapidly through point A to D, jumping from 0 to 110.88 thousand hectares on arable land and to 23.36 thousand hectares on unutilized agricultural land. When we compare the contribution of two kinds of land resources, the arable land serves as the main source for sorghum plantation. At point B, C, and D, the sown area of sorghum on this land is 106 times, 27 times, and five times as much as on unutilized agricultural land, respectively. However, facing the target of producing both sufficient grains and biomass, the potential of arable land resources for sorghum plantation is exhausted at point C. Once past that point, the task of sorghum cultivation will be handed over to unutilized agricultural land. This maneuver can also be demonstrated through the changes of the land shadow price. Given the assumption that crop patterns on arable land are bind to their historical sown areas, the crop mix on this kind of land resources is not granted much flexibility, thus leading to a stable shadow price. In comparison, without such constraints, the introduction of sweet sorghum can significantly change the crop pattern on unutilized agricultural land. The higher the biomass price is, the more the unutilized agricultural land is allocated for sweet sorghum cultivation. Therefore, the shadow price increases along with the growth of the biomass price. As a result of the spillover effect by the dramatic change of crop patterns on heavy & average soil, the sown area of crops on other soil types fluctuates slightly.

**Table 8:** Usage of arable land and unutilized agricultural land resources on standard supply curve (Unit: 10^3^ ha)

Illustrative points	Arable land resources										
	Total sown area	Heavy & average				Light			Very light		
		Crop rotation Total	Monoculture		Price^b^ (€/ha)	Crop rotation Total	Monoculture Total	Price (€/ha)	Crop rotation Total	Monoculture Total	Price (€/ha)
			Total	Sorghum^a^							
A	895.85	73.28	482.62	–	162.50	95.81	96.88	189.02	129.35	17.90	193.02
B	895.85	69.46	486.45	33.90	162.50	93.33	99.37	189.02	129.34	17.90	193.02
C	895.86	57.78	498.14	110.58	162.50	89.48	103.21	189.02	129.64	17.61	193.02
D	895.86	57.27	498.65	110.88	162.50	95.83	96.86	189.02	129.83	17.42	193.02

Illustrative points	Unutilized agricultural land resources										
	Total sown area	Heavy & average				Light			Very light		
		Crop rotation Total	Monoculture		Price (€/ha)	Crop rotation Total	Monoculture Total	Price (€/ha)	Crop rotation Total	Monoculture Total	Price (€/ha)
			Total	Sorghum							
A	101.63	5.15	36.79	–	392.81	2.70	18.76	375.89	2.30	35.92	153.00
B	101.70	4.79	37.17	0.32	405.46	2.62	18.84	393.40	1.89	36.38	153.64
C	101.80	0.98	40.98	4.10	446.94	2.63	18.83	444.16	3.12	35.26	155.63
D	101.79	–	41.96	23.36	588.10	2.50	18.96	533.03	2.90	35.46	156.07

a According to the model assumption, sweet sorghum is planted monoculturally on Heavy & average soil.

b The column of price stands for the rental price of the corresponding land resources. Its unit is €/ha, while the units of all other columns are thousand hectare.

**Figure 9:**
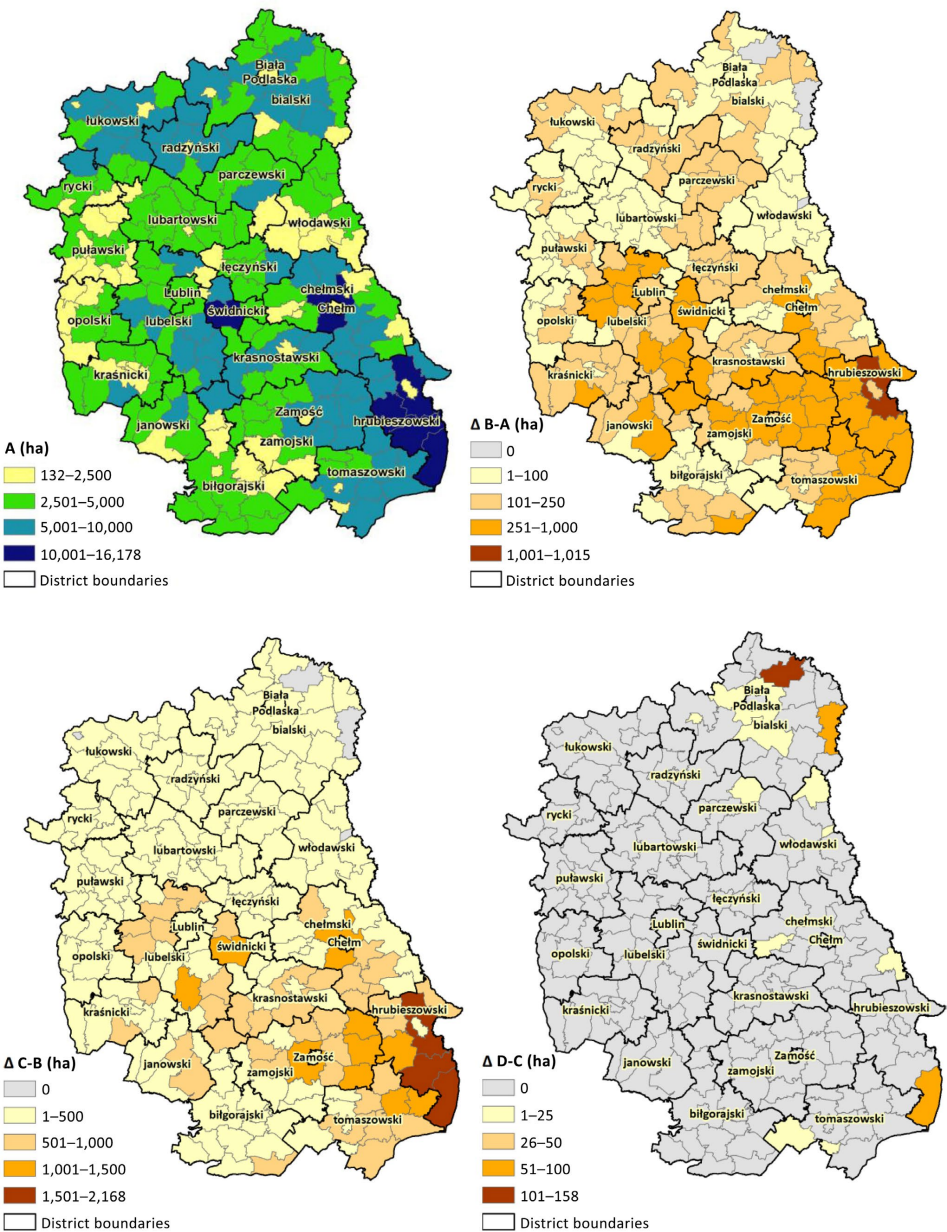
Arable land use change in Lubelski voivodeship at four illustrative points. Notes: Map A presents the absolute value of the cultivation area of conventional crops at point A. Maps of ΔB‐A, ΔC‐B, and ΔD‐C illustrate the change of the sown areas between two neighbouring points, describing the expansion of sweet sorghum plantation

To spatially illustrate the introduction process of sweet sorghum and dynamically disclose the land use competition, we draw the development of the cultivated area of aggregate conventional crops and sweet sorghum at the LAU level at the aforementioned four points in Figure 9.

Our model reveals that the decreased sown area of conventional crops is mainly switched to the sorghum cultivation, indicating the occurrence of land use competition between food security and biomass production. At point A, due to the low price of sweet sorghum, the revenue from this crop is not competitive at all to its conventional counterparts. All arable land resources are allocated to cultivate conventional crops. Their crop residues are the only potential biomass sources. Łukowski, Radzyński, and Bialski *powiat*s located on the north, Lubelski, Świdnicki, Chełmski, and Krasnostawski *powiat*s located in the center, and Zamojski, Hrubieszowski, and Tomaszowski *powiat*s located on the southeast are the strongholds of conventional crops. When biomass price rises, sweet sorghum starts to penetrate to the central and south‐eastern *powiat*s, while the strongholds on the north retain their resistance to the large‐scale introduction of sorghum. Moving from point B to C, the expansion of sorghum follows the same feature as in the previous phase. The central and south‐eastern strongholds of conventional crops continue providing the majority of arable land resources to the sorghum cultivation. However, this trend suspends at point D, where the available land in those strongholds are depleted. Alternatively, the originally highly resistant *gmina*s lying at the north‐eastern part of Bialski *powiat* start to introduce sorghum. The high biomass price may play a role here. In the end, sweet sorghum is scattered in all 213 *gmina*s of the Lubelski voivodeship.

#### 3.2.4 Composition of biomass supply

**Figure 10:**
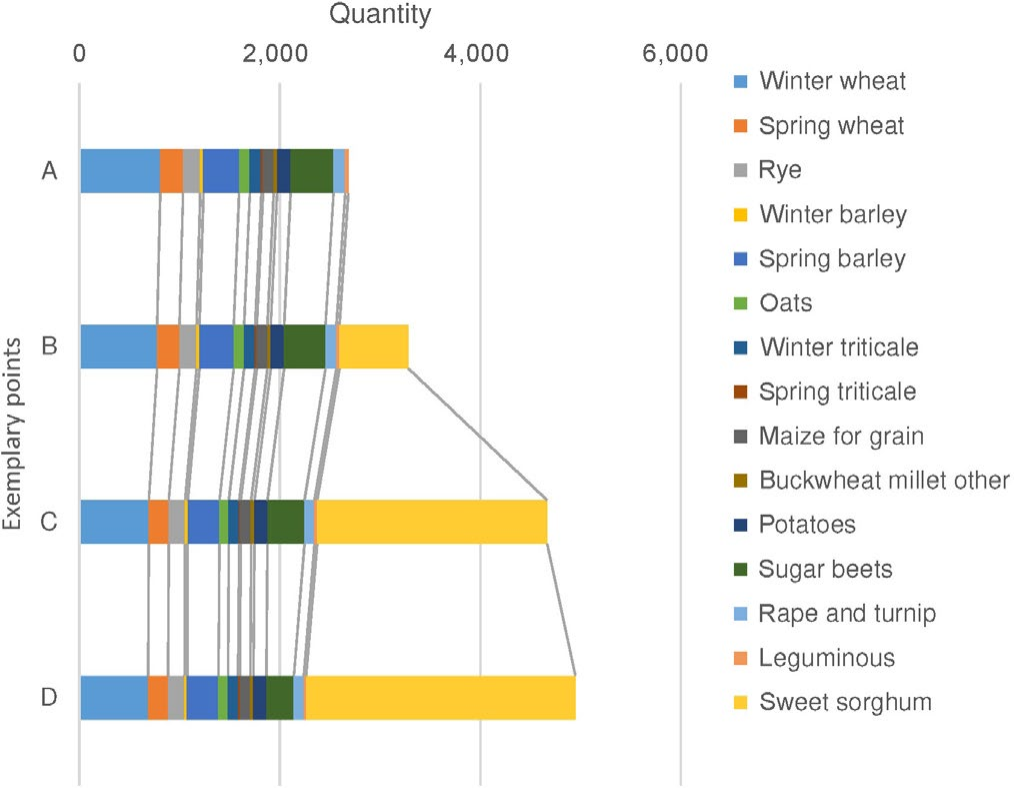
Composition of biomass potential at four exemplary points

In the meantime, we have drawn Figure 10 to present the biomass supply by sources at four illustrative points.

In line with the overarching trend expressed by the biomass supply curve in Figure 7, a significant increase in biomass output is realized by the introduction of sweet sorghum, denoting its indispensable role in securing the biomass potential. Without the participation of the DEC, total biomass production staggers at around 2.68 million tonnes. The crop residues from winter wheat lead a long way ahead, constituting more than 30% of the total supply at point A, followed by residues from sugar beet, spring barley, and spring wheat (15.96%, 13.23%, and 8.47%, respectively, see Figure 10). The introduction of sorghum greatly pulls up the total biomass production level, amounting from 3.28 million tonnes at point B, 4.67 million tonnes at point C to 4.95 million tonnes at point D. Correspondingly, its share in total biomass provision rockets from 0 at point A to 20.86% at point B, and further to 49.16% at point C. In the end, it alone supplies 54.26% of the total biomass feedstock at point D. In this process, the outputs from sugar beets, winter wheat, and spring barley curtail sharply, while the contributions of spring triticale, maize for grain, and buckwheat millet and other are hardly affected. This implies that the land resources for sorghum cultivation are mainly offered by leading conventional crops, that is, sugar beets, winter wheat, and spring barley.

## 4 DISCUSSION

### 4.1 Life cycle of sweet sorghum introduction process

Akin to a typical industry life cycle, the four illustrative points identified on the supply curve can be interpreted as the thresholds of separating four development stages of the sweet sorghum introduction (Figure 7). Between point A and B, namely the “startup” stage, sweet sorghum starts to appear in the areas where it is most profitable. In this stage, the traditional crop patterns on arable land are largely maintained and there is no usage of unutilized agricultural land due to its relatively high reclamation cost. Between point B and C, the production of sweet sorghum enters a “growth” phase with the feature of highly elastic supply curve. The accruement of sorghum output mainly comes from the arable land at the cost of mild contraction of conventional crop cultivation on the same piece of land. Between point C and D, this industry welcomes the “shakeout” era with a stiff supply curve featuring a rapid growth of market price against a weak increase of sorghum output. That implies the depletion of arable land resources. Interestingly, above point D, this phase is succeed by “revival” instead of “maturity” stage, which is commonly defined in the traditional life cycle concept. The reason here is the role of unutilized agricultural land functioning as candidate resources. Once the price exceeds 21.10 €/ha, presumably a level sufficiently offsetting the reclamation cost of these resources, the upward momentum of sorghum cultivation is picked up and pushes the technical potential of sweet sorghum to 7.5 million tonnes. By contrast, the economic potential lying at 6 million tonnes can be achieved at reasonable price ranging between 11.70 and 16.40 €/tonne.

From the perspective of the theory of industry life cycle, the growth stage is a vital phase for the industry development. New products slowly draw attention from customers and profitability starts to rise, which attracts more producers to join. At the level of companies, their revenue continues to rise and start generating positive cash flows and profits as product revenue and costs break‐even. This also holds true for sweet sorghum introduction. Attracting enough farmers to cultivate sorghum voluntarily with a least disturbance on local socioeconomic and natural environment is the overarching policy target in this phase.

### 4.2 Land use competition

However, the land use competition between food crops and energy crops is widely observed in the stage of growth, which is clearly demonstrated by our simulation results (Figure 9). Our model proposes two ways to alleviate this issue, which may cast some lights on local land use policy design.

The first solution is to increase the crop yield of sweet sorghum. As we suppose, the green mass yield of sorghum is 50 tonnes/ha. However, considering the results from a field experiment conducted in Osiny, Poland, its yield can be between 74.0 and 94.6 tonnes/ha under different nitrogen fertilization levels (Księżak et al., 2012). Furthermore, thanks to its trait of drought tolerance, this species can better adapt to the climate change in Poland than conventional crops, where a decrease in runoff of rivers as well as soil moisture in summer months during pronounced precipitation deficits is projected (Kundzewicz & Matczak, 2012). Although introducing sweet sorghum can be beneficial to alleviate climate change impacts, its effectiveness is largely depended on the performance of regular crops. As illustrated by our sensitivity analysis, crop yield loss under climate change will plague the introduction of sweet sorghum.

An alternative way is to introduce the unutilized agricultural land resources, which has been touched on by other studies (Gerssen‐Gondelach, Wicke, Borzęcka‐Walker, Pudełko, & Faaij, 2016; Pudełko et al., 2012). Since the reform of Polish agriculture in 1990, the use of marginal land and part of small agricultural parcels located on good soil becomes unprofitable, leading to a large scale of farmland abandonment. The model discusses the possibility of using this kind of resources to replace the arable land for energy crops plantation. However, the simulation results reveal that this proposal may not work when the market price of sorghum is too low to compensate the reclamation cost of these resources, that is, below 21.10 €/ha in our case. Therefore, we suggest introducing particular subsidies on these abandoned land resources within the local CAP scheme.

### 4.3 Model extension

Our model suggests the economic biomass potential in Lubelski region ranging between 4.31 and 8.11 million tonnes. This result generally agrees with the research of Rozakis et al. (2013), which pins the number at 6.62 million tonnes. However, the composition of biomass feedstock in two studies is divergent. In ours, while sweet sorghum is considered as a promising source, other purposes of crop residues are beyond the scope, such as animal feeding and bedding, as well as substrates for mushroom production. To fix this defect, the livestock sector is to be integrated to the model. In such a way, the source and quantity of livestock manure, another important source for biogas plants, can be simulated. Lack of access to detailed field data also contributes to the divergence to some extent. For example, two levels of crop pattern data are used in our model. Although the data at the LAU level collected from 1996, 2002, and 2010, agricultural survey can provide the details of crop mix in each *gmina*, they largely fail to reflect the policy impacts in recent years. To fill this gap, we have to introduce the annual dataset on crop patterns at the NUTS‐2 level. However, individual crop rotations and the corresponding crop yields as well as the cultivation cost cannot be directly derived from such low resolution data. Upon our simplified assumptions, the model delivers the land opportunity cost of heavy and average, light, and very light soil at 162.50, 189.02, and 193.02 €/ha, respectively, while the corresponding rental prices reported in 2017 are 199.78, 181.03, and 148.90 €/ha (Statistics Poland, 2019). Although the land opportunity costs and rental prices are of the same order of magnitude, there are mismatches in the relative value of the three soil types. In order to accurately evaluate the existing crop rotations and propose suitable ones under the envisaged CAP2020, our model should be calibrated by field data, which can correctly reflect the differentiated effects of crop rotations and soil types on crop yields and cultivation cost.

## 5 CONCLUSION

As the first comprehensive and high resolution study of its kind to focus on the sweet sorghum introduction in Poland to our knowledge, this model‐driven research dynamically illustrates the introduction process, explores the impacts on land use change and assesses the technical and economic potential of sweet sorghum.

In this paper, we have developed a spatial agent dynamic model of agricultural land use for Poland. Each aggregate farmer at the LAU level is treated as an independent agent. Like other mathematical optimization models, agents in our research are assumed to make their own decisions on adjusting crop patterns so as to maximize the total welfare of the agricultural sector and fulfill the constraints set at LAU and NUTS‐2 level in the meantime. The introduction of sweet sorghum is realized by the farmers’ response to its continuously increasing price.

To tailor the model to the transitional feature of the Polish agriculture sector in Poland, we have applied the model to the Lubelski voivodeship. Under the current framework, we have examined 15 conventional crops and sweet sorghum, 213 *gmina*s, three soil types, six typical crop rotations, two‐layer historical crop patterns, and both arable land and unutilized agricultural land resources. Based on our simulation results, five conclusions can be made. (a) A high variability of land use change of conventional crops is expected. (b) At relatively low biomass price, crop residues from conventional crops provide the whole biomass supply. At the price of 8.20 €/tonne, sorghum is to be appear for the first time in the Lubelski voivodeship, and gradually dominates the supply of biomass along with the price growth. (c) The economic and technical potential of sweet sorghum is estimated to 6 and 7.5 million tonnes, respectively. (d) Similar to other industry life cycles, the introduction process of sweet sorghum will experience “startup,” “growth,” “shakeout,” and “revival” phases consecutively. (e) Land use competition between conventional crops and sweet sorghum is expected to occur in the growth phase. Improving the yield of sorghum and reclaiming unutilized agricultural land may alleviate this conflict, but special focus on climate change is needed. Our analysis demonstrates that climate change can extensively constrain the introduction of sweet sorghum.

This model has provided us many insightful results. Before we further apply this model to other voivodeships or to the state level of Poland, it is advisable to use field data to further calibrate the model, integrate the livestock sector, and evaluate the climate change impacts.

## APPENDIX A

Model Framework

**Indices**

**Table:**

Term	Definition and elements
*allt*	time horizon /1999‐2050/
*ht(allt)*	historical year involved in observation /1999‐2017/
*t(allt)*	projection year /2018‐2050/
*r*	213 regions in Lubelskie on NUT 5 level /0601011‐0664011/
*cc*	combined crops/wheat, rye, barley, oats, triticale, potatoes, sugar‐beets, rape‐and‐turnip/
*fc*	conventional crops/winter‐wheat, spring‐wheat, rye, winter‐barley, spring‐barley, oats, winter‐triticale, spring‐triticale, maize‐for‐grain, maize‐for‐forage, buckwheat‐millet‐other, potato, sugar‐beet, rape‐and‐turnip, leguminous‐edible/
*nfpc(fc)*	conventional crops excluding forage crops/winter‐wheat, spring‐wheat, rye, winter‐barley, spring‐barley, oats, winter‐triticale, spring‐triticale, maize‐for‐grain, potato, sugar‐beet, rape‐and‐turnip, leguminous‐edible/
*ec*	energy crops/sweet sorghum/
*grains*	grains/winter‐wheat, spring‐wheat, rye, winter‐barley, spring‐barley, oats, winter‐triticale, spring‐triticale, maize‐for‐grain, maize‐for‐forage, buckwheat‐millet‐other, potatoes, sugar‐beets, rape‐and‐turnip, leguminous‐edible/
*biomass*	bioenergy feedstock/straw, sweet sorghum/
*s*	policy scenarios/s1/
*a*	age classes/a1,a2,…,a15/
*st*	soil type/heavy‐average, light, very‐light/
*cr*	crop rotation pattern/s‐sw‐ww‐wb, m‐sw‐ww‐wb, l‐ww‐ww‐wb, ra‐ww‐ww‐wb, p‐sb‐wt‐c‐wt, o‐ry‐c, mono/

**Parameters**

**Table:**

Term	Definition and elements
$y_{r,t,fc,pr,st,cr}^{conventional\;crop}$	yield of conventional crop (10^3^ t/10^3^ ha)
$y_{ec,biomass,a}^{energy\;crop}$	yield of energy crop (10^3^ t/10^3^ ha)
$price_{t,pr}$	product price (10^6^ PLN/10^3^ t)
$ps_{t,pr,s}$	price subsidy of products (10^6^ PLN/10^3^ t)
$sub_{r,t,fc,s}^{conventional\;crop}$	land subsidy for conventional crops (10^6^ PLN/10^3^ ha)
$sub_{r,t,ec,s}^{energy\;crop}$	land subsidy for perennial crops (10^6^ PLN/10^3^ ha)
$h_{r,ht,fc}$	historical cultivation data at *gmina* level (10^3^ ha)
$b_{r}^{\mathrm{land}}$	arable land area for each *gmina* (10^3^ ha)
$his_{ht,fc}$	historical cultivation data at voivodeship level (10^3^ ha)
$unub_{r,st}^{\mathrm{land}}$	total unutilized land area (10^3^ ha)
$k_{\mathrm{ec}}$	expected lifespan of energy crops (years)
$dema_{t,grains}^{\mathrm{grains}}$	demand of grains (10^3^t)
*discount*	discount rate (unitless)
$sh_{r,st}$	share of each soil type
$c_{r,t,fc}^{conventional\;crop}$	plantation cost of conventional crops (10^6^PLN/10^3^ ha)
$c_{r,t,ec,a}^{energy\;crop}$	plantation cost of energy crops (10^6^PLN/10^3^ ha)

**Decision variables**

**Nonnegative variables**

**Table:**

Term	Definition and elements
$LAND_{r,t,fc,st,cr}^{conventional\;crop}$	cultivated area for food crops on arable lands (10^3^ ha)
$LAND_{r,t,ec,a,st,cr}^{energy\;crop}$	cultivated area for energy crops on arable lands (10^3^ ha)
$UNULAND_{r,t,fc,st,cr}^{conventional\;crop}$	cultivated area for food crops on unutilized land (10^3^ ha)
$UNULAND_{r,t,ec,a,st,cr}^{energy\;crop}$	cultivated area for energy crops on unutilized lands (10^3^ ha)
$CMIXP_{t,ht}$	weights of historical observation at voivodeship level

**Objective function**

(A.1) $$\begin{array}{c} \text{Max WELF}=\sum_{t}{\left(1+discount\right)}^{-t}\\ \cdot \left\{\begin{array}{c} \sum_{r,fc,grains,st,cr}\left[y_{r,t,fc,grains}^{conventional\;crop}\cdot \left(LAND_{r,t,fc,st,cr}^{conventional\;crop}+UNULAND_{r,t,fc,st,cr}^{conventional\;crop}\right)\cdot \left(price_{t,grains}+ps_{t,grains,s}\right)\right]\\ +\sum_{r,fc,biomass,st,cr}\left[y_{r,t,fc,biomass}^{conventional\;crop}\cdot \left(LAND_{r,t,fc,st,cr}^{conventional\;crop}+UNULAND_{r,t,fc,st,cr}^{conventional\;crop}\right)\cdot ps_{t,biomass,s}\right]\\ +\sum_{r,fc,ec,biomass,a,st,cr}\left[y_{ec,biomass,a}^{energy\;crop}\cdot \left(LAND_{r,t,ec,a,st,cr}^{energy\;crop}+UNULAND_{r,t,ec,a,st,cr}^{energy\;crop}\right)\cdot \left(price_{t,biomass}+ps_{t,biomass,s}\right)\right]\\ +\sum_{r,fc,st,cr}\left[\left(LAND_{r,t,fc,st,cr}^{conventional\;crop}+UNULAND_{r,t,fc,st,cr}^{conventional\;crop}\right)\cdot sub_{r,t,fc,s}^{conventional\;crop}\right]\\ +\sum_{r,ec,a,st,cr}\left[\left(LAND_{r,t,ec,a,st,cr}^{energy\;crop}+UNULAND_{r,t,ec,a,st,cr}^{energy\;crop}\right)\cdot sub_{r,t,ec,s}^{energy\;crop}\right] \end{array}\right\}\\ -\sum_{t}{\left(1+discount\right)}^{-t}\\ \cdot \left\{\begin{array}{c} \sum_{r,fc,st,cr}\left[c_{r,t,fc}^{conventional\;crop}\cdot \left(LAND_{r,t,fc,st,cr}^{conventional\;crop}+UNULAND_{r,t,fc,st,cr}^{conventional\;crop}\right)\right]\\ +\sum_{r,ec,a,st,cr}\left[c_{r,t,ec,a}^{energy\;crop}\cdot \left(LAND_{r,t,ec,a,st,cr}^{energy\;crop}+UNULAND_{r,t,ec,a,st,cr}^{energy\;crop}\right)\right] \end{array}\right\}\\ \forall s. \end{array}$$

**Subject to**

**Arable land resource endowments constraint**

(A.2) $$\sum_{fc,cr}LAND_{r,t,fc,st,cr}^{conventional\;crop}+\sum_{ec,a,cr}LAND_{r,t,ec,a,st,cr}^{energy\;crop}\leq b_{r}^{lan\;d}\cdot sh_{r,st}\;\forall r,t,st.$$

**Unutilized land resource endowments constraint**

(A.3) $$\sum_{fc,cr}UNULAND_{r,t,fc,st,cr}^{conventional\;crop}+\sum_{ec,a,cr}UNULAND_{r,t,ec,a,st,cr}^{energy\;crop}\leq unub_{r,st}^{lan\;d}\;\forall r,t,st.$$

**Crop mix constraint at voivodeship level**

(A.4) $$-\sum_{\mathrm{ht}}\left(his_{ht,cc}\cdot CMIXP_{t,ht}\right)+\sum_{r,st,cr}LAND_{r,t,fc,st,cr}^{conventional\;crop}=0\;\forall t,fc,cc.$$

**Crop mix weight constraint**

(A.5) $$2\cdot \sum_{ht\in 1999-2011}CMIXP_{t,ht}-\sum_{ht\in 2012-2017}CMIXP_{t,ht}\leq 0\;\forall t.$$

**Sown area change constraint**

(A.6) $$\sum_{st,cr}LAND_{r,t,nfpc,st,cr}^{conventional\;crop}+\sum_{st,cr}UNULAND_{r,t,nfpc,st,cr}^{conventional\;crop}\leq 2\cdot h_{r,\text{"}2010\text{"},nfpc}\;\forall r,t,nfpc.$$

**Crop rotation constraint**

1. ½ sugar beet, spring wheat, winter wheat, winter barley

(A.7) $$\begin{array}{c} 2\cdot LAND_{r,t,\text{"}sugar-beets\text{"},\text{"}heavy-average\text{"},\text{"}s-sw-ww-wb\text{"}}^{conventional\;crop}\leq LAND_{r,t,\text{"}spring-wheat\text{"},\text{"}heavy-average\text{"},\text{"}s-sw-ww-wb\text{"}}^{conventional\;crop}\\ LAND_{r,t,\text{"}spring-wheat\text{"},\text{"}heavy-average\text{"},\text{"}s-sw-ww-wb\text{"}}^{conventional\;crop}\leq LAND_{r,t,\text{"}winter-wheat\text{"},\text{"}heavy-average\text{"},\text{"}s-sw-ww-wb\text{"}}^{conventional\;crop}\\ LAND_{r,t,\text{"}winter-wheat\text{"},\text{"}heavy-average\text{"},\text{"}s-sw-ww-wb\text{"}}^{conventional\;crop}\leq LAND_{r,t,\text{"}winter-barley\text{"},\text{"}heavy-average\text{"},\text{"}s-sw-ww-wb\text{"}}^{conventional\;crop}\\ 1/2\cdot LAND_{r,t,\text{"}winter-barley\text{"},\text{"}heavy-average\text{"},\text{"}s-sw-ww-wb\text{"}}^{conventional\;crop}\leq LAND_{r,t,\text{"}sugar-beets\text{"},\text{"}heavy-average\text{"},\text{"}s-sw-ww-wb\text{"}}^{conventional\;crop}. \end{array}$$

1. ½ maize, spring wheat, winter wheat, winter barley

(A.8) $$\begin{array}{c} 2\cdot (LAND_{r,t,\text{"}maize-for-grain\text{"},\text{"}heavy-average\text{"},\text{"}m-sw-ww-wb\text{"}}^{conventional\;crop}+LAND_{r,t,\text{"}maize-for-forage\text{"},\text{"}heavy-average\text{"},\text{"}m-sw-ww-wb\text{"}}^{conventional\;crop})\\ \;\leq LAND_{r,t,\text{"}spring-wheat\text{"},\text{"}heavy-average\text{"},\text{"}m-sw-ww-wb\text{"}}^{conventional\;crop}\\ LAND_{r,t,\text{"}spring-wheat\text{"},\text{"}heavy-average\text{"},\text{"}m-sw-ww-wb\text{"}}^{conventional\;crop}\leq LAND_{r,t,\text{"}winter-wheat\text{"},\text{"}heavy-average\text{"},\text{"}m-sw-ww-wb\text{"}}^{conventional\;crop}\\ LAND_{r,t,\text{"}winter-wheat\text{"},\text{"}heavy-average\text{"},\text{"}m-sw-ww-wb\text{"}}^{conventional\;crop}\leq LAND_{r,t,\text{"}winter-barley\text{"},\text{"}heavy-average\text{"},\text{"}m-sw-ww-wb\text{"}}^{conventional\;crop}\\ 1/2\cdot LAND_{r,t,\text{"}winter-barley\text{"},\text{"}heavy-average\text{"},\text{"}m-sw-ww-wb\text{"}}^{conventional\;crop}\\ \;\leq LAND_{r,t,\text{"}maize-for-grain\text{"},\text{"}heavy-average\text{"},\text{"}m-sw-ww-wb\text{"}}^{conventional\;crop}+LAND_{r,t,\text{"}maize-for-forage\text{"},\text{"}heavy-average\text{"},\text{"}m-sw-ww-wb\text{"}}^{conventional\;crop}\\ \forall r,t. \end{array}$$

1. ½ leguminous edible, winter wheat, winter wheat, winter barley

(A.9) $$\begin{array}{c} 4\cdot LAND_{r,t,\text{"}leguminous-edible\text{"},\text{"}heavy-average\text{"},\text{"}l-ww-ww-wb\text{"}}^{conventional\;crop}\leq LAND_{r,t,\text{"}winter-wheat\text{"},\text{"}heavy-average\text{"},\text{"}l-w-ww-wb\text{"}}^{conventional\;crop}\\ 1/2\cdot LAND_{r,t,\text{"}winter-wheat\text{"},\text{"}heavy-average\text{"},\text{"}l-ww-ww-wb\text{"}}^{conventional\;crop}\leq LAND_{r,t,\text{"}winter-barley\text{"},\text{"}heavy-average\text{"},\text{"}l-ww-ww-wb\text{"}}^{conventional\;crop}\\ 1/2\cdot LAND_{r,t,\text{"}winter-barley\text{"},\text{"}heavy-average\text{"},\text{"}l-ww-ww-wb\text{"}}^{conventional\;crop}\leq LAND_{r,t,\text{"}leguminous-edible\text{"},\text{"}heavy-average\text{"},\text{"}l-ww-ww-wb\text{"}}^{conventional\;crop}\\ \forall r,t. \end{array}$$

1. ½ rape and turnip, winter wheat, winter wheat, winter barley

(A.10) $$\begin{array}{c} 4\cdot LAND_{r,t,\text{"}rape-and-turnip\text{"},\text{"}heavy-average\text{"},\text{"}ra-ww-ww-wb\text{"}}^{conventional\;crop}\leq LAND_{r,t,\text{"}winter-wheat\text{"},\text{"}heavy-average\text{"},\text{"}ra-ww-ww-wb\text{"}}^{conventional\;crop}\\ 1/2\cdot LAND_{r,t,\text{"}winter-wheat\text{"},\text{"}heavy-average\text{"},\text{"}ra-ww-ww-wb\text{"}}^{conventional\;crop}\leq LAND_{r,t,\text{"}winter-barley\text{"},\text{"}heavy-average\text{"},\text{"}ra-ww-ww-wb\text{"}}^{conventional\;crop}\\ 1/2\cdot LAND_{r,t,\text{"}winter-barley\text{"},\text{"}heavy-average\text{"},\text{"}ra-ww-ww-wb\text{"}}^{conventional\;crop}\leq LAND_{r,t,\text{"}rape-and-turnip\text{"},\text{"}heavy-average\text{"},\text{"}ra-ww-ww-wb\text{"}}^{conventional\;crop}\\ \forall r,t. \end{array}$$

1. potatoes, spring barley, winter triticale, cereal mixture, winter triticale

(A.11) $$\begin{array}{c} LAND_{r,t,\text{"}potatoes\text{"},\text{"}heavy-average\text{"}/\text{"}light\text{"},\text{"}p-sb-wt-c-wt\text{"}}^{conventional\;crop}\leq LAND_{r,t,\text{"}spring-barley\text{"},\text{"}heavy-average\text{"}/\text{"}light\text{"},\text{"}p-sb-wt-c-wt\text{"}}^{conventional\;crop}\\ LAND_{r,t,\text{"}spring-barley\text{"},\text{"}heavy-average\text{"}/\text{"}light\text{"},\text{"}p-sb-wt-c-wt\text{"}}^{conventional\;crop}\leq LAND_{r,t,\text{"}winter-triticale\text{"},\text{"}heavy-average\text{"}/\text{"}light\text{"},\text{"}p-sb-wt-c-wt\text{"}}^{conventional\;crop}\\ LAND_{r,t,\text{"}oats\text{"},\text{"}heavy-average\text{"}/\text{"}light\text{"},\text{"}p-sb-wt-c-wt\text{"}}^{conventional\;crop}+LAND_{r,t,\text{"}spring-wheat\text{"},\text{"}heavy-average\text{"}/\text{"}light\text{"},\text{"}p-sb-wt-c-wt\text{"}}^{conventional\;crop}\\ \;\leq 1/2\cdot LAND_{r,t,\text{"}winter-triticale\text{"},\text{"}heavy-average\text{"}/\text{"}light\text{"},\text{"}p-sb-wt-c-wt\text{"}}^{conventional\;crop}\\ 1/2\cdot LAND_{r,t,\text{"}winter-triticale\text{"},\text{"}heavy-average\text{"}/\text{"}light\text{"},\text{"}p-sb-wt-c-wt\text{"}}^{conventional\;crop}\leq LAND_{r,t,\text{"}potatoes\text{"},\text{"}heavy-average\text{"}/\text{"}light\text{"},\text{"}p-sb-wt-c-wt\text{"}}^{conventional\;crop}\\ \forall r,t. \end{array}$$

1. oats, rye, cereal mixture

(A.12) $$\begin{array}{c} LAND_{r,t,\text{"}rye\text{"},\text{"}very-light\text{"}/\text{"}light\text{"},\text{"}o-ry-c\text{"}}^{conventional\;crop}\leq LAND_{r,t,\text{"}oats\text{"},\text{"}very-light\text{"}/\text{"}light\text{"},\text{"}o-ry-c\text{"}}^{conventional\;crop}\\ LAND_{r,t,\text{"}spring-barley\text{"},\text{"}very-light\text{"}/\text{"}light\text{"},\text{"}o-ry-c\text{"}}^{conventional\;crop}+LAND_{r,t,\text{"}spring-wheat\text{"},\text{"}very-light\text{"}/\text{"}light\text{"},\text{"}o-ry-c\text{"}}^{conventional\;crop}\\ \;\leq LAND_{r,t,\text{"}rye\text{"},\text{"}very-light\text{"}/\text{"}light\text{"},\text{"}o-ry-c\text{"}}^{conventional\;crop}\\ LAND_{r,t,\text{"}oats\text{"},\text{"}very-light\text{"}/\text{"}light\text{"},\text{"}o-ry-c\text{"}}^{conventional\;crop}\leq 2\cdot LAND_{r,t,\text{"}rye\text{"},\text{"}very-light\text{"}/\text{"}light\text{"},\text{"}o-ry-c\text{"}}^{conventional\;crop}\\ \forall r,t. \end{array}$$

**Energy crop consistency constraint on arable land**

(A.13) $$-LAND_{r,t-1,ec,a-1,st,cr}^{energy\;crop}+LAND_{r,t,ec,a,st,cr}^{energy\;crop}{\leq 0|}_{1<a\leq k_{\mathrm{ec}}}\;\forall r,t,ec,a,st,cr.$$

**Energy crop consistency constraint on unutilized land**

(A.14) $$-UNULAND_{r,t-1,ec,a-1,st,cr}^{energy\;crop}+UNULAND_{r,t,ec,a,st,cr}^{energy\;crop}{\leq 0|}_{1<a\leq k_{\mathrm{ec}}}\;\forall r,t,ec,a,st,cr.$$

**Food security constraint**

(A.15) $$dema_{t,grains}^{\mathrm{grains}}-\sum_{r,fc,st,cr}\left[y_{r,t,fc,grains}^{conventional\;crop}\cdot \left(LAND_{r,t,fc,st,cr}^{conventional\;crop}+UNULAND_{r,t,fc,st,cr}^{conventional\;crop}\right)\right]\;\leq 0\;\forall t,grains.$$

**Sorghum share constraint**

(A.16) $$\sum_{ec,a,cr}LAND_{r,t,ec,a,st,cr}^{energy\;crop}\leq 0.2\cdot b_{r}^{lan\;d}\cdot sh_{r,st}\;\forall r,t,st.$$
